# Tetra­aqua­bis­(μ_2_-4,4′-bipyridine)­dodeca­kis­(μ_2_-2-methyl­prop-2-enoato)octa­kis­(2-methyl­prop-2-enoato)tetra­ytterbium(III)tetra­zinc(II)

**DOI:** 10.1107/S1600536810034215

**Published:** 2010-09-04

**Authors:** Bin Wu, Chengxin Zhao

**Affiliations:** a Department of Chemistry, Zhejiang Sci-Tech University, Hangzhou 310018, People’s Republic of China

## Abstract

The asymmetric unit of the title compound, [Yb_4_Zn_4_(C_4_H_5_O_2_)_20_(C_10_H_8_N_2_)_2_(H_2_O)_4_], contains half of a centrosymmetric octa­nuclear mol­ecule in which each Zn^II^ ion is four-coordinated by three O atoms from three 2-methyl­prop-2-enoate (*L*) ligands and one N atom from a 4,4′-bipyridine (bipy) ligand in a distorted pyramidal geometry. The two independent Yb^III^ ions, each coordinated by eight O atoms in an irregular geometry, exhibit different coordination environments, *viz.* one water mol­ecule, five bridging bidentate and one chelating bidentate carboxyl­ate groups for one Yb^III^ ion, and one water mol­ecule, three bridging bidentate and two chelating bidentate carboxyl­ate groups for the other Yb^III^ ion. In the crystal structure, inter­molecular O—H⋯O and C—H⋯O hydrogen bonds help to establish the packing.

## Related literature

For the crystal structures of analogous complexes, see: Wu *et al.* (2003 ▶, 2004 ▶). For details of the preparation of Yb*L*_3_ (*L* = methacrylate), see: Lu *et al.* (1995 ▶).
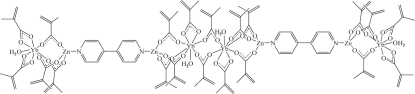


## Experimental

### 

#### Crystal data

[Yb_4_Zn_4_(C_4_H_5_O_2_)_20_(C_10_H_8_N_2_)_2_(H_2_O)_4_]
*M* *_r_* = 1519.84
Triclinic, 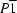

*a* = 13.398 (3) Å
*b* = 14.880 (3) Å
*c* = 16.408 (3) Å
α = 64.06 (3)°
β = 89.55 (3)°
γ = 85.54 (3)°
*V* = 2931.5 (10) Å^3^
*Z* = 2
Mo *K*α radiation
μ = 4.05 mm^−1^
*T* = 293 K
0.26 × 0.22 × 0.10 mm

#### Data collection

Rigaku R-AXIS RAPID diffractometer
Absorption correction: multi-scan (*ABSCOR*; Higashi, 1995 ▶) *T*_min_ = 0.381, *T*_max_ = 0.673
22308 measured reflections
10712 independent reflections
8847 reflections with *I* > 2σ(*I*)
*R*_int_ = 0.055

#### Refinement

*R*[*F* ^2^ > 2σ(*F* ^2^)] = 0.030
*wR*(*F* ^2^) = 0.066
*S* = 1.01
10712 reflections
714 parameters
H-atom parameters constrained
Δρ_max_ = 0.57 e Å^−3^
Δρ_min_ = −0.87 e Å^−3^

### 

Data collection: *RAPID-AUTO* (Rigaku, 1998 ▶); cell refinement: *RAPID-AUTO*; data reduction: *CrystalStructure* (Rigaku/MSC, 2002 ▶); program(s) used to solve structure: *SHELXS97* (Sheldrick, 2008 ▶); program(s) used to refine structure: *SHELXL97* (Sheldrick, 2008 ▶); molecular graphics: *ORTEP-3* (Farrugia, 1997 ▶); software used to prepare material for publication: *SHELXL97*.

## Supplementary Material

Crystal structure: contains datablocks global, I. DOI: 10.1107/S1600536810034215/cv2750sup1.cif

Structure factors: contains datablocks I. DOI: 10.1107/S1600536810034215/cv2750Isup2.hkl

Additional supplementary materials:  crystallographic information; 3D view; checkCIF report

## Figures and Tables

**Table 1:** Hydrogen-bond geometry (Å, °)

*D*—H⋯*A*	*D*—H	H⋯*A*	*D*⋯*A*	*D*—H⋯*A*
O1—H11⋯O4^i^	0.93	2.01	2.795 (4)	142
O1—H12⋯O2^i^	0.72	2.30	2.903 (5)	142
O22—H221⋯O19^ii^	0.71	2.06	2.758 (4)	169
C19—H19*A*⋯O3	0.96	2.54	3.339 (8)	141
C22—H22⋯O18^iii^	0.93	2.45	3.297 (5)	152
C24—H24⋯O5^iv^	0.93	2.52	3.435 (6)	169
C27—H27⋯O18^iii^	0.93	2.40	3.330 (5)	175
C30—H30⋯O5^iv^	0.93	2.42	3.256 (6)	149

Symmetry codes: (i) 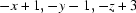; (ii) 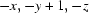; (iii) 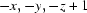; (iv) 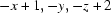.

**Fig. 1.**
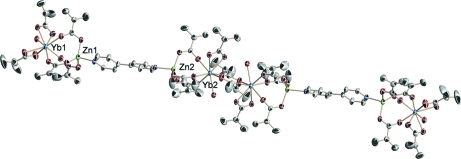
View of (I) showing the atomic numbering of the Yb and Zn atoms. The unlabelled atoms are related with the labelled ones by symmetry operation (-x, 1-y, -z). Displacement ellipsoids are drawn at the 20% probability level. H atoms have been omitted for clarity.

## supplementary crystallographic information

### Comment

The study of heterometallic complexes containing *d*-transition metal and lanthanide(III) cations connected by bridging ligands is being actively pursued because of their relevance in solid-state technology and as models for magnetic studies. As a contribution to a structural study of heterometallic complexes containing d-transition metal and rare-earth(III) cations, herewith we report the synthesis and crystal structure of the title compound, (I).

The crystal structure of the title Yb—Zn complex is similar to the known crystal structures of the Gd—Zn and Tb—Zn complexes (Wu *et al.*, 2003, 2004). As shown in Figure 1, the complex consists of a discrete octanuclear molecule, which possesses a symmetry center between the two ytterbium (III) ions. All bridges between the metallic ions are formed by two kinds of ligands. Two Yb^III^ ions are linked by two carboxylato groups. Zn^II^ and Yb^III^ ions are bridged by three carboxylato groups, and two Zn^II^ ions are linked by 4,4'-bipy molecule. There are two different coordination circumstances for the ytterbium ions in the titled complex. One ytterbium [Yb1] is coordinated by eight O atoms from one water molecule, three bridging bidentate and two chelating bidentate carboxylato groups. The other [Yb2] is also coordinated by eight O atoms, but from five bridging bidentate, one chelating bidentate carboxylato groups and a water molecule. The coordination sphere around each ytterbium (III) ion is irregular. Each zinc(II) ion is four-coordinated by three O atoms from three bridging carboxylato groups and one N atom from 4,4'-bipy group. The coordination polyhedron is a distorted tetrahedron. As is the common case, the carboxylato groups in the title complex serve as chelating or bridging bidentate ligands. The separations of Yb1···Zn1, Zn1···Zn2, Yb2···Zn2 and Yb2···Yb2* (symmetry code: -*x*, 1 - *y*, -*z*) are 3.870 (1), 11.189 (1), 4.012 (1) and 4.745 (1) Å, respectively. In the crystal, O—H···O and C—H···O hydrogen bonds (Table 1) help to establish the packing.

### Experimental

Yb*L*_3_ (H*L* = CH_2_C(CH_3_)COOH), has been prepared following the known procedure (Lu *et al.*, 1995). Yb*L*_3_ (860 mg, 2.0 mmol; HL=CH_2_C(Me)CO_2_H) and Zn(NO_3_)_2_.6H2O (240 mg, 0.8 mmol) were dissolved in H_2_O (20 ml) and adjusted pH=4.1 with HL (0.1 *M*). EtOH (3 ml) solution of 4,4'-bipy (60 mg, 0.4 mmol) was added to the mixed solution with stirring. After filtration, the filtrate was allowed to stand at room temperature and single crystals suitable for X-ray work were obtained after 2 weeks.

### Refinement

All H-atoms were placed in idealized locations with C–H distances 0.93 - 0.96 Å and refined as riding, with  *U*_iso_(H) = 1.2 or 1.5 times *U*_eq_(C). The water' H-atoms were located on a difference map and refined as riding, with  *U*_iso_(H) = 0.05 Å^-2^.

### Crystal data

**Table:**

[Yb_4_Zn_4_(C_4_H_5_O_2_)_20_(C_10_H_8_N_2_)_2_(H_2_O)_4_]	*Z* = 2
*M**_r_* = 1519.84	*F*(000) = 1504
Triclinic, *P*1	*D*_x_ = 1.722 Mg m^−^^3^
Hall symbol: -P 1	Mo *K*α radiation, λ = 0.71069 Å
*a* = 13.398 (3) Å	Cell parameters from 14142 reflections
*b* = 14.880 (3) Å	θ = 1.5–27.5°
*c* = 16.408 (3) Å	µ = 4.05 mm^−^^1^
α = 64.06 (3)°	*T* = 293 K
β = 89.55 (3)°	Platelet, colourless
γ = 85.54 (3)°	0.26 × 0.22 × 0.10 mm
*V* = 2931.5 (10) Å^3^	

### Data collection

**Table:**

Rigaku R-AXIS RAPID diffractometer	10712 independent reflections
Radiation source: fine-focus sealed tube	8847 reflections with *I* > 2σ(*I*)
graphite	*R*_int_ = 0.055
Detector resolution: 10.00 pixels mm^-1^	θ_max_ = 25.7°, θ_min_ = 2.1°
ω scans	*h* = −16→15
Absorption correction: multi-scan (*ABSCOR*; Higashi, 1995)	*k* = −18→18
*T*_min_ = 0.381, *T*_max_ = 0.673	*l* = −19→19
22308 measured reflections	

### Refinement

**Table:**

Refinement on *F*^2^	Secondary atom site location: difference Fourier map
Least-squares matrix: full	Hydrogen site location: inferred from neighbouring sites
*R*[*F*^2^ > 2σ(*F*^2^)] = 0.030	H-atom parameters constrained
*wR*(*F*^2^) = 0.066	*w* = 1/[σ^2^(*F*_o_^2^) + (0.0305*P*)^2^ + 4.1373*P*] where *P* = (*F*_o_^2^ + 2*F*_c_^2^)/3
*S* = 1.01	(Δ/σ)_max_ = 0.001
10712 reflections	Δρ_max_ = 0.56 e Å^−^^3^
714 parameters	Δρ_min_ = −0.87 e Å^−^^3^
0 restraints	Extinction correction: *SHELXL97* (Sheldrick, 2008)
Primary atom site location: structure-invariant direct methods	

### Special details

**Table:**

Geometry. All e.s.d.'s (except the e.s.d. in the dihedral angle between two l.s. planes) are estimated using the full covariance matrix. The cell e.s.d.'s are taken into account individually in the estimation of e.s.d.'s in distances, angles and torsion angles; correlations between e.s.d.'s in cell parameters are only used when they are defined by crystal symmetry. An approximate (isotropic) treatment of cell e.s.d.'s is used for estimating e.s.d.'s involving l.s. planes.
Refinement. Refinement of *F*^2^ against ALL reflections. The weighted *R*-factor *wR* and goodness of fit *S* are based on *F*^2^, conventional *R*-factors *R* are based on *F*, with *F* set to zero for negative *F*^2^. The threshold expression of *F*^2^ > σ(*F*^2^) is used only for calculating *R*-factors(gt) *etc*. and is not relevant to the choice of reflections for refinement. *R*-factors based on *F*^2^ are statistically about twice as large as those based on *F*, and *R*- factors based on ALL data will be even larger.

### Fractional atomic coordinates and isotropic or equivalent isotropic displacement parameters (Å^2^)

**Table:**

	*x*	*y*	*z*	*U*_iso_*/*U*_eq_	
Yb1	0.508466 (13)	−0.300505 (13)	1.355980 (11)	0.02897 (6)	
Yb2	0.004178 (13)	0.362808 (13)	0.146271 (11)	0.02782 (6)	
Zn1	0.37941 (4)	−0.14090 (4)	1.12076 (3)	0.03521 (12)	
Zn2	0.05248 (4)	0.20108 (4)	0.41429 (3)	0.03255 (12)	
O1	0.4222 (2)	−0.4461 (2)	1.41376 (19)	0.0388 (7)	
O2	0.4903 (3)	−0.3484 (3)	1.5144 (2)	0.0486 (9)	
O3	0.5883 (3)	−0.2324 (3)	1.4400 (2)	0.0607 (11)	
O4	0.6321 (2)	−0.4369 (2)	1.4082 (2)	0.0422 (8)	
O5	0.6751 (2)	−0.2949 (2)	1.3032 (2)	0.0447 (8)	
O6	0.3458 (2)	−0.2437 (2)	1.3379 (2)	0.0440 (8)	
O7	0.2706 (2)	−0.1904 (3)	1.2026 (2)	0.0430 (8)	
O8	0.4883 (2)	−0.3320 (2)	1.23534 (19)	0.0403 (7)	
O9	0.4813 (3)	−0.2225 (3)	1.0916 (2)	0.0519 (9)	
O10	0.5116 (2)	−0.1372 (2)	1.2531 (2)	0.0443 (8)	
O11	0.4339 (2)	−0.0260 (2)	1.1270 (2)	0.0435 (8)	
O12	0.1318 (2)	0.1243 (2)	0.36295 (19)	0.0409 (7)	
O13	0.1356 (2)	0.2590 (2)	0.23113 (19)	0.0369 (7)	
O14	0.0948 (3)	0.3303 (2)	0.39321 (19)	0.0431 (8)	
O15	0.0481 (3)	0.4258 (2)	0.2485 (2)	0.0465 (8)	
O16	−0.0884 (2)	0.1816 (2)	0.41191 (19)	0.0433 (8)	
O17	−0.0915 (2)	0.2780 (2)	0.2644 (2)	0.0444 (8)	
O18	−0.0054 (2)	0.2014 (2)	0.14016 (19)	0.0375 (7)	
O19	0.0683 (2)	0.3203 (2)	0.03094 (19)	0.0387 (7)	
O20	−0.1357 (2)	0.3832 (2)	0.05811 (19)	0.0423 (8)	
O21	−0.1044 (2)	0.5200 (2)	−0.0638 (2)	0.0394 (7)	
O22	−0.0976 (2)	0.5133 (2)	0.12861 (19)	0.0423 (8)	
N1	0.2990 (3)	−0.0752 (3)	0.9994 (2)	0.0352 (8)	
N2	0.0968 (3)	0.1303 (3)	0.5499 (2)	0.0311 (8)	
C1	0.5478 (3)	−0.2838 (4)	1.5125 (3)	0.0394 (11)	
C2	0.5691 (4)	−0.2710 (5)	1.5954 (4)	0.0592 (15)	
C3	0.6218 (6)	−0.1922 (8)	1.5880 (6)	0.129 (4)	
H3A	0.6349	−0.1827	1.6391	0.155*	
H3B	0.6444	−0.1485	1.5318	0.155*	
C4	0.5330 (10)	−0.3390 (9)	1.6756 (5)	0.166 (5)	
H4A	0.5645	−0.4046	1.6903	0.250*	
H4B	0.4619	−0.3395	1.6694	0.250*	
H4C	0.5474	−0.3203	1.7231	0.250*	
C5	0.6960 (3)	−0.3867 (4)	1.3531 (3)	0.0393 (11)	
C6	0.7951 (4)	−0.4363 (4)	1.3467 (3)	0.0503 (13)	
C7	0.8043 (5)	−0.5372 (5)	1.3754 (5)	0.080 (2)	
H7A	0.8657	−0.5693	1.3724	0.096*	
H7B	0.7493	−0.5742	1.3981	0.096*	
C8	0.8749 (5)	−0.3738 (5)	1.3112 (5)	0.092 (2)	
H8A	0.9332	−0.4131	1.3070	0.138*	
H8B	0.8898	−0.3439	1.3506	0.138*	
H8C	0.8557	−0.3220	1.2520	0.138*	
C9	0.2693 (3)	−0.2205 (3)	1.2876 (3)	0.0336 (10)	
C10	0.1684 (4)	−0.2281 (3)	1.3292 (3)	0.0446 (12)	
C11	0.1633 (5)	−0.2555 (5)	1.4193 (4)	0.0707 (18)	
H11A	0.1013	−0.2596	1.4461	0.085*	
H11B	0.2218	−0.2702	1.4544	0.085*	
C12	0.0810 (4)	−0.2040 (5)	1.2696 (4)	0.0681 (17)	
H12A	0.0217	−0.2019	1.3023	0.102*	
H12B	0.0776	−0.2542	1.2481	0.102*	
H12C	0.0857	−0.1398	1.2190	0.102*	
C13	0.5172 (3)	−0.3026 (4)	1.1559 (3)	0.0378 (10)	
C14	0.5975 (4)	−0.3629 (4)	1.1343 (3)	0.0527 (13)	
C15	0.6227 (6)	−0.4576 (5)	1.1916 (4)	0.083 (2)	
H15A	0.6745	−0.4939	1.1781	0.099*	
H15B	0.5887	−0.4878	1.2453	0.099*	
C16	0.6496 (6)	−0.3128 (7)	1.0490 (5)	0.128 (4)	
H16A	0.7204	−0.3301	1.0597	0.192*	
H16B	0.6362	−0.2415	1.0259	0.192*	
H16C	0.6263	−0.3340	1.0056	0.192*	
C17	0.4874 (3)	−0.0490 (3)	1.1985 (3)	0.0355 (10)	
C18	0.5211 (3)	0.0348 (3)	1.2153 (3)	0.0403 (11)	
C19	0.5493 (5)	0.0133 (5)	1.3069 (4)	0.0733 (19)	
H19A	0.5922	−0.0478	1.3329	0.110*	
H19B	0.4903	0.0058	1.3421	0.110*	
H19C	0.5841	0.0673	1.3067	0.110*	
C20	0.5244 (4)	0.1279 (4)	1.1437 (4)	0.0552 (14)	
H20A	0.5456	0.1807	1.1532	0.066*	
H20B	0.5054	0.1381	1.0857	0.066*	
C21	0.2442 (3)	−0.1296 (4)	0.9731 (3)	0.0433 (12)	
H21	0.2348	−0.1949	1.0147	0.052*	
C22	0.2005 (3)	−0.0942 (3)	0.8872 (3)	0.0422 (12)	
H22	0.1634	−0.1351	0.8719	0.051*	
C23	0.2130 (3)	0.0040 (3)	0.8243 (3)	0.0305 (9)	
C24	0.2674 (3)	0.0619 (3)	0.8531 (3)	0.0350 (10)	
H24	0.2752	0.1286	0.8140	0.042*	
C25	0.3093 (3)	0.0202 (3)	0.9393 (3)	0.0363 (10)	
H25	0.3463	0.0595	0.9567	0.044*	
C26	0.1713 (3)	0.0460 (3)	0.7295 (3)	0.0300 (9)	
C27	0.0940 (3)	0.0057 (3)	0.7037 (3)	0.0347 (10)	
H27	0.0655	−0.0503	0.7463	0.042*	
C28	0.0598 (3)	0.0494 (3)	0.6141 (3)	0.0352 (10)	
H28	0.0086	0.0210	0.5980	0.042*	
C29	0.1710 (3)	0.1685 (4)	0.5750 (3)	0.0417 (12)	
H29	0.1974	0.2251	0.5312	0.050*	
C30	0.2106 (4)	0.1290 (3)	0.6619 (3)	0.0429 (12)	
H30	0.2636	0.1577	0.6752	0.052*	
C31	0.1603 (3)	0.1684 (3)	0.2823 (3)	0.0311 (9)	
C32	0.2290 (3)	0.1077 (4)	0.2499 (3)	0.0407 (11)	
C33	0.2556 (5)	0.0025 (4)	0.3141 (5)	0.078 (2)	
H33A	0.2943	−0.0305	0.2840	0.118*	
H33B	0.1956	−0.0307	0.3361	0.118*	
H33C	0.2943	0.0000	0.3641	0.118*	
C34	0.2629 (4)	0.1506 (5)	0.1663 (3)	0.0586 (15)	
H34A	0.3061	0.1136	0.1455	0.070*	
H34B	0.2435	0.2175	0.1287	0.070*	
C35	0.0932 (3)	0.4102 (3)	0.3202 (3)	0.0349 (10)	
C36	0.1514 (4)	0.4908 (4)	0.3226 (4)	0.0472 (12)	
C37	0.1837 (6)	0.4796 (5)	0.4124 (5)	0.092 (2)	
H37A	0.2340	0.4246	0.4384	0.137*	
H37B	0.1273	0.4669	0.4512	0.137*	
H37C	0.2107	0.5402	0.4060	0.137*	
C38	0.1730 (6)	0.5662 (5)	0.2448 (5)	0.089 (2)	
H38A	0.2106	0.6161	0.2450	0.107*	
H38B	0.1505	0.5691	0.1901	0.107*	
C39	−0.1328 (3)	0.2246 (3)	0.3347 (3)	0.0338 (10)	
C40	−0.2415 (4)	0.2103 (4)	0.3314 (3)	0.0540 (14)	
C41	−0.2866 (5)	0.1437 (6)	0.4176 (4)	0.091 (3)	
H41A	−0.3555	0.1380	0.4065	0.136*	
H41B	−0.2825	0.1715	0.4603	0.136*	
H41C	−0.2508	0.0785	0.4419	0.136*	
C42	−0.2898 (5)	0.2550 (6)	0.2518 (4)	0.087 (2)	
H42A	−0.3570	0.2456	0.2473	0.104*	
H42B	−0.2566	0.2958	0.2005	0.104*	
C43	0.0392 (3)	0.2328 (3)	0.0665 (3)	0.0379 (10)	
C44	0.0579 (4)	0.1662 (4)	0.0197 (3)	0.0479 (12)	
C45	0.0655 (5)	0.0604 (4)	0.0732 (4)	0.0715 (17)	
H45A	0.0719	0.0275	0.0343	0.107*	
H45B	0.0065	0.0414	0.1083	0.107*	
H45C	0.1233	0.0410	0.1132	0.107*	
C46	0.0688 (7)	0.2109 (6)	−0.0730 (4)	0.099 (3)	
H46A	0.0819	0.1715	−0.1035	0.119*	
H46B	0.0632	0.2805	−0.1049	0.119*	
C47	−0.1625 (3)	0.4572 (3)	−0.0149 (3)	0.0321 (9)	
C48	−0.2703 (4)	0.4716 (4)	−0.0459 (3)	0.0473 (12)	
C49	−0.3324 (5)	0.3943 (6)	0.0011 (4)	0.090 (2)	
H49A	−0.3686	0.4084	0.0454	0.136*	
H49B	−0.2922	0.3318	0.0308	0.136*	
H49C	−0.3789	0.3898	−0.0410	0.136*	
C50	−0.3050 (5)	0.5593 (5)	−0.1184 (5)	0.102 (3)	
H50A	−0.3716	0.5689	−0.1380	0.123*	
H50B	−0.2618	0.6090	−0.1480	0.123*	
H11	0.3881	−0.4567	1.4662	0.050*	
H12	0.4449	−0.4878	1.4071	0.050*	
H221	−0.0977	0.5582	0.0876	0.050*	
H222	−0.0758	0.5249	0.1699	0.050*	

### Atomic displacement parameters (Å^2^)

**Table:**

	*U*^11^	*U*^22^	*U*^33^	*U*^12^	*U*^13^	*U*^23^
Yb1	0.03336 (11)	0.02672 (11)	0.02086 (9)	−0.00583 (8)	−0.00164 (7)	−0.00436 (7)
Yb2	0.03188 (10)	0.02507 (10)	0.02023 (9)	−0.00485 (7)	−0.00078 (7)	−0.00371 (7)
Zn1	0.0385 (3)	0.0378 (3)	0.0230 (2)	−0.0006 (2)	−0.00541 (19)	−0.0078 (2)
Zn2	0.0398 (3)	0.0327 (3)	0.0220 (2)	−0.0039 (2)	−0.00316 (19)	−0.0088 (2)
O1	0.0408 (17)	0.0384 (18)	0.0302 (16)	−0.0134 (14)	0.0035 (13)	−0.0071 (14)
O2	0.063 (2)	0.052 (2)	0.0346 (18)	−0.0232 (18)	0.0095 (15)	−0.0192 (16)
O3	0.083 (3)	0.064 (2)	0.041 (2)	−0.037 (2)	0.0079 (18)	−0.0232 (18)
O4	0.0396 (17)	0.0379 (18)	0.0347 (17)	−0.0034 (14)	0.0049 (13)	−0.0026 (14)
O5	0.0398 (18)	0.0340 (19)	0.052 (2)	−0.0090 (15)	0.0091 (15)	−0.0103 (16)
O6	0.0442 (18)	0.051 (2)	0.0347 (17)	0.0089 (16)	−0.0061 (14)	−0.0183 (16)
O7	0.0372 (17)	0.054 (2)	0.0322 (17)	−0.0081 (15)	0.0002 (13)	−0.0132 (15)
O8	0.0495 (19)	0.0397 (18)	0.0297 (16)	−0.0083 (15)	0.0003 (13)	−0.0126 (14)
O9	0.062 (2)	0.057 (2)	0.0233 (16)	0.0184 (18)	−0.0027 (14)	−0.0088 (16)
O10	0.057 (2)	0.0262 (17)	0.0407 (18)	−0.0075 (15)	−0.0019 (15)	−0.0059 (14)
O11	0.0485 (19)	0.0404 (19)	0.0361 (17)	−0.0053 (15)	−0.0078 (14)	−0.0113 (15)
O12	0.057 (2)	0.0312 (17)	0.0310 (17)	−0.0022 (15)	0.0075 (14)	−0.0107 (14)
O13	0.0365 (16)	0.0326 (17)	0.0351 (16)	0.0003 (13)	−0.0049 (13)	−0.0092 (14)
O14	0.065 (2)	0.0320 (17)	0.0274 (16)	−0.0075 (15)	−0.0102 (14)	−0.0076 (14)
O15	0.070 (2)	0.0345 (18)	0.0327 (17)	0.0013 (16)	−0.0168 (15)	−0.0130 (15)
O16	0.0419 (18)	0.053 (2)	0.0277 (16)	−0.0123 (16)	−0.0020 (13)	−0.0099 (15)
O17	0.0420 (18)	0.049 (2)	0.0309 (17)	−0.0106 (15)	0.0097 (13)	−0.0066 (15)
O18	0.0474 (18)	0.0314 (17)	0.0314 (16)	−0.0107 (14)	0.0021 (13)	−0.0103 (13)
O19	0.0533 (19)	0.0304 (17)	0.0279 (15)	−0.0091 (15)	0.0050 (13)	−0.0077 (13)
O20	0.0411 (18)	0.0414 (19)	0.0314 (17)	−0.0079 (15)	−0.0094 (13)	−0.0030 (15)
O21	0.0435 (18)	0.0332 (17)	0.0366 (17)	−0.0138 (14)	0.0048 (14)	−0.0092 (14)
O22	0.055 (2)	0.0366 (18)	0.0280 (16)	0.0050 (15)	0.0004 (14)	−0.0085 (14)
N1	0.036 (2)	0.037 (2)	0.0253 (18)	0.0001 (16)	−0.0086 (14)	−0.0073 (16)
N2	0.0373 (19)	0.0298 (19)	0.0221 (17)	−0.0039 (16)	−0.0028 (14)	−0.0073 (15)
C1	0.039 (3)	0.042 (3)	0.036 (3)	0.003 (2)	−0.0077 (19)	−0.017 (2)
C2	0.058 (3)	0.085 (4)	0.044 (3)	−0.001 (3)	−0.008 (2)	−0.038 (3)
C3	0.132 (7)	0.216 (11)	0.118 (7)	−0.097 (8)	0.043 (6)	−0.132 (8)
C4	0.278 (15)	0.189 (11)	0.049 (5)	−0.084 (11)	0.021 (7)	−0.057 (6)
C5	0.038 (2)	0.046 (3)	0.034 (2)	−0.010 (2)	0.0030 (19)	−0.016 (2)
C6	0.042 (3)	0.055 (3)	0.049 (3)	0.005 (2)	0.003 (2)	−0.019 (3)
C7	0.061 (4)	0.057 (4)	0.096 (5)	0.018 (3)	0.015 (3)	−0.013 (4)
C8	0.057 (4)	0.088 (5)	0.120 (6)	−0.009 (4)	0.037 (4)	−0.035 (5)
C9	0.037 (2)	0.026 (2)	0.037 (2)	−0.0049 (19)	0.0021 (19)	−0.0122 (19)
C10	0.040 (3)	0.032 (3)	0.060 (3)	−0.009 (2)	0.012 (2)	−0.017 (2)
C11	0.069 (4)	0.081 (5)	0.053 (4)	−0.001 (3)	0.029 (3)	−0.023 (3)
C12	0.036 (3)	0.074 (4)	0.098 (5)	−0.007 (3)	0.008 (3)	−0.041 (4)
C13	0.039 (2)	0.044 (3)	0.028 (2)	−0.003 (2)	−0.0026 (18)	−0.014 (2)
C14	0.059 (3)	0.058 (3)	0.036 (3)	0.019 (3)	−0.007 (2)	−0.019 (3)
C15	0.109 (6)	0.055 (4)	0.070 (4)	0.022 (4)	0.013 (4)	−0.018 (3)
C16	0.116 (6)	0.146 (8)	0.061 (5)	0.067 (6)	0.034 (4)	−0.001 (5)
C17	0.031 (2)	0.035 (3)	0.035 (2)	−0.0074 (19)	0.0042 (18)	−0.010 (2)
C18	0.037 (2)	0.034 (3)	0.047 (3)	−0.007 (2)	−0.001 (2)	−0.015 (2)
C19	0.110 (5)	0.058 (4)	0.056 (4)	−0.015 (4)	−0.016 (3)	−0.027 (3)
C20	0.064 (3)	0.040 (3)	0.054 (3)	−0.012 (3)	−0.001 (3)	−0.012 (3)
C21	0.047 (3)	0.038 (3)	0.032 (2)	−0.012 (2)	−0.007 (2)	−0.002 (2)
C22	0.045 (3)	0.040 (3)	0.031 (2)	−0.015 (2)	−0.0101 (19)	−0.004 (2)
C23	0.030 (2)	0.032 (2)	0.022 (2)	−0.0040 (18)	−0.0004 (16)	−0.0053 (17)
C24	0.043 (3)	0.030 (2)	0.024 (2)	−0.005 (2)	−0.0034 (18)	−0.0044 (18)
C25	0.041 (2)	0.036 (3)	0.028 (2)	−0.003 (2)	−0.0085 (18)	−0.0102 (19)
C26	0.030 (2)	0.032 (2)	0.022 (2)	−0.0007 (18)	−0.0030 (16)	−0.0071 (18)
C27	0.042 (2)	0.028 (2)	0.025 (2)	−0.0108 (19)	−0.0036 (17)	−0.0028 (18)
C28	0.040 (2)	0.034 (2)	0.030 (2)	−0.013 (2)	−0.0044 (18)	−0.0106 (19)
C29	0.046 (3)	0.041 (3)	0.024 (2)	−0.017 (2)	−0.0023 (18)	0.0015 (19)
C30	0.049 (3)	0.042 (3)	0.025 (2)	−0.020 (2)	−0.0111 (19)	0.0004 (19)
C31	0.034 (2)	0.028 (2)	0.027 (2)	−0.0023 (18)	−0.0046 (17)	−0.0093 (18)
C32	0.041 (3)	0.039 (3)	0.043 (3)	−0.001 (2)	0.000 (2)	−0.020 (2)
C33	0.099 (5)	0.036 (3)	0.089 (5)	0.005 (3)	0.035 (4)	−0.018 (3)
C34	0.050 (3)	0.071 (4)	0.044 (3)	0.018 (3)	0.004 (2)	−0.018 (3)
C35	0.041 (2)	0.030 (2)	0.032 (2)	−0.0011 (19)	−0.0019 (18)	−0.012 (2)
C36	0.053 (3)	0.034 (3)	0.058 (3)	−0.003 (2)	−0.002 (2)	−0.023 (2)
C37	0.109 (6)	0.075 (5)	0.102 (5)	−0.023 (4)	−0.039 (4)	−0.046 (4)
C38	0.136 (7)	0.050 (4)	0.095 (5)	−0.045 (4)	0.041 (5)	−0.039 (4)
C39	0.037 (2)	0.036 (2)	0.033 (2)	−0.011 (2)	0.0035 (18)	−0.019 (2)
C40	0.051 (3)	0.070 (4)	0.039 (3)	−0.028 (3)	0.000 (2)	−0.017 (3)
C41	0.068 (4)	0.138 (7)	0.056 (4)	−0.058 (5)	0.012 (3)	−0.024 (4)
C42	0.059 (4)	0.126 (6)	0.061 (4)	−0.040 (4)	−0.010 (3)	−0.022 (4)
C43	0.041 (3)	0.035 (3)	0.034 (2)	0.001 (2)	−0.0076 (19)	−0.012 (2)
C44	0.055 (3)	0.045 (3)	0.051 (3)	−0.003 (2)	0.000 (2)	−0.028 (3)
C45	0.087 (5)	0.054 (4)	0.083 (4)	−0.011 (3)	0.007 (4)	−0.039 (3)
C46	0.186 (9)	0.070 (5)	0.054 (4)	0.003 (5)	0.008 (5)	−0.040 (4)
C47	0.031 (2)	0.033 (2)	0.031 (2)	−0.0052 (19)	−0.0022 (17)	−0.0126 (19)
C48	0.038 (3)	0.057 (3)	0.039 (3)	−0.005 (2)	−0.011 (2)	−0.013 (2)
C49	0.059 (4)	0.104 (6)	0.082 (5)	−0.039 (4)	−0.016 (3)	−0.011 (4)
C50	0.054 (4)	0.085 (5)	0.105 (6)	−0.006 (4)	−0.032 (4)	0.017 (4)

### Geometric parameters (Å, °)

**Table:**

Yb1—O8	2.247 (3)	C12—H12A	0.9600
Yb1—O6	2.249 (3)	C12—H12B	0.9600
Yb1—O10	2.276 (3)	C12—H12C	0.9600
Yb1—O3	2.342 (3)	C13—C14	1.486 (7)
Yb1—O1	2.346 (3)	C14—C15	1.325 (8)
Yb1—O4	2.361 (3)	C14—C16	1.468 (8)
Yb1—O5	2.383 (3)	C15—H15A	0.9300
Yb1—O2	2.392 (3)	C15—H15B	0.9300
Yb1—H12	2.7404 (8)	C16—H16A	0.9600
Yb2—O21^i^	2.224 (3)	C16—H16B	0.9600
Yb2—O17	2.250 (3)	C16—H16C	0.9600
Yb2—O13	2.271 (3)	C17—C18	1.492 (6)
Yb2—O20	2.295 (3)	C18—C20	1.373 (6)
Yb2—O15	2.349 (3)	C18—C19	1.438 (7)
Yb2—O19	2.378 (3)	C19—H19A	0.9600
Yb2—O22	2.433 (3)	C19—H19B	0.9600
Yb2—O18	2.461 (3)	C19—H19C	0.9600
Yb2—H222	2.7437 (7)	C20—H20A	0.9300
Zn1—O7	1.934 (3)	C20—H20B	0.9300
Zn1—O9	1.950 (4)	C21—C22	1.386 (6)
Zn1—O11	1.954 (3)	C21—H21	0.9300
Zn1—N1	2.061 (3)	C22—C23	1.395 (6)
Zn2—O14	1.929 (3)	C22—H22	0.9300
Zn2—O16	1.935 (3)	C23—C24	1.399 (6)
Zn2—O12	1.945 (3)	C23—C26	1.493 (5)
Zn2—N2	2.071 (3)	C24—C25	1.375 (5)
O1—H11	0.927	C24—H24	0.9300
O1—H12	0.720	C25—H25	0.9300
O2—C1	1.267 (5)	C26—C30	1.389 (5)
O3—C1	1.246 (5)	C26—C27	1.392 (6)
O4—C5	1.267 (5)	C27—C28	1.385 (5)
O5—C5	1.257 (5)	C27—H27	0.9300
O6—C9	1.249 (5)	C28—H28	0.9300
O7—C9	1.265 (5)	C29—C30	1.374 (6)
O8—C13	1.249 (5)	C29—H29	0.9300
O9—C13	1.259 (5)	C30—H30	0.9300
O10—C17	1.241 (5)	C31—C32	1.492 (6)
O11—C17	1.277 (5)	C32—C34	1.328 (7)
O12—C31	1.265 (5)	C32—C33	1.471 (7)
O13—C31	1.256 (5)	C33—H33A	0.9600
O14—C35	1.265 (5)	C33—H33B	0.9600
O15—C35	1.245 (5)	C33—H33C	0.9600
O16—C39	1.269 (5)	C34—H34A	0.9300
O17—C39	1.235 (5)	C34—H34B	0.9300
O18—C43	1.256 (5)	C35—C36	1.495 (6)
O19—C43	1.265 (5)	C36—C38	1.328 (8)
O20—C47	1.252 (5)	C36—C37	1.473 (8)
O21—C47	1.253 (5)	C37—H37A	0.9600
O21—Yb2^i^	2.224 (3)	C37—H37B	0.9600
O22—H221	0.712	C37—H37C	0.9600
O22—H222	0.830	C38—H38A	0.9300
N1—C21	1.335 (6)	C38—H38B	0.9300
N1—C25	1.346 (5)	C39—C40	1.493 (6)
N2—C28	1.334 (5)	C40—C42	1.324 (7)
N2—C29	1.333 (5)	C40—C41	1.484 (7)
C1—C2	1.485 (6)	C41—H41A	0.9600
C2—C4	1.375 (10)	C41—H41B	0.9600
C2—C3	1.375 (9)	C41—H41C	0.9600
C3—H3A	0.9300	C42—H42A	0.9300
C3—H3B	0.9300	C42—H42B	0.9300
C4—H4A	0.9600	C43—C44	1.501 (7)
C4—H4B	0.9600	C44—C46	1.379 (8)
C4—H4C	0.9600	C44—C45	1.424 (7)
C5—C6	1.495 (7)	C45—H45A	0.9600
C6—C7	1.358 (8)	C45—H45B	0.9600
C6—C8	1.421 (8)	C45—H45C	0.9600
C7—H7A	0.9300	C46—H46A	0.9300
C7—H7B	0.9300	C46—H46B	0.9300
C8—H8A	0.9600	C47—C48	1.503 (6)
C8—H8B	0.9600	C48—C50	1.376 (8)
C8—H8C	0.9600	C48—C49	1.405 (7)
C9—C10	1.500 (6)	C49—H49A	0.9600
C10—C11	1.353 (7)	C49—H49B	0.9600
C10—C12	1.450 (7)	C49—H49C	0.9600
C11—H11A	0.9300	C50—H50A	0.9300
C11—H11B	0.9300	C50—H50B	0.9300
			
O8—Yb1—O6	87.66 (12)	O7—C9—C10	116.8 (4)
O8—Yb1—O10	85.68 (11)	C11—C10—C12	123.6 (5)
O6—Yb1—O10	77.08 (13)	C11—C10—C9	118.9 (5)
O8—Yb1—O3	154.05 (12)	C12—C10—C9	117.6 (5)
O6—Yb1—O3	108.06 (14)	C10—C11—H11A	120.0
O10—Yb1—O3	78.23 (12)	C10—C11—H11B	120.0
O8—Yb1—O1	76.44 (11)	H11A—C11—H11B	120.0
O6—Yb1—O1	75.57 (12)	C10—C12—H12A	109.5
O10—Yb1—O1	147.73 (11)	C10—C12—H12B	109.5
O3—Yb1—O1	126.74 (11)	H12A—C12—H12B	109.5
O8—Yb1—O4	86.29 (12)	C10—C12—H12C	109.5
O6—Yb1—O4	149.31 (11)	H12A—C12—H12C	109.5
O10—Yb1—O4	132.26 (11)	H12B—C12—H12C	109.5
O3—Yb1—O4	89.59 (14)	O8—C13—O9	122.7 (4)
O1—Yb1—O4	73.76 (11)	O8—C13—C14	120.0 (4)
O8—Yb1—O5	78.57 (12)	O9—C13—C14	117.3 (4)
O6—Yb1—O5	151.91 (11)	C15—C14—C16	121.5 (6)
O10—Yb1—O5	77.56 (12)	C15—C14—C13	120.8 (5)
O3—Yb1—O5	78.16 (13)	C16—C14—C13	117.6 (5)
O1—Yb1—O5	123.51 (11)	C14—C15—H15A	120.0
O4—Yb1—O5	54.73 (11)	C14—C15—H15B	120.0
O8—Yb1—O2	149.68 (11)	H15A—C15—H15B	120.0
O6—Yb1—O2	87.13 (12)	C14—C16—H16A	109.5
O10—Yb1—O2	122.00 (12)	C14—C16—H16B	109.5
O3—Yb1—O2	54.35 (11)	H16A—C16—H16B	109.5
O1—Yb1—O2	73.33 (11)	C14—C16—H16C	109.5
O4—Yb1—O2	83.13 (12)	H16A—C16—H16C	109.5
O5—Yb1—O2	116.77 (12)	H16B—C16—H16C	109.5
O8—Yb1—H12	68.90 (8)	O10—C17—O11	122.5 (4)
O6—Yb1—H12	86.96 (9)	O10—C17—C18	120.0 (4)
O10—Yb1—H12	150.56 (8)	O11—C17—C18	117.6 (4)
O3—Yb1—H12	130.74 (9)	C20—C18—C19	123.2 (5)
O1—Yb1—H12	13.64 (8)	C20—C18—C17	119.1 (4)
O4—Yb1—H12	62.82 (8)	C19—C18—C17	117.6 (4)
O5—Yb1—H12	110.10 (8)	C18—C19—H19A	109.5
O2—Yb1—H12	81.01 (8)	C18—C19—H19B	109.5
O21^i^—Yb2—O17	156.53 (12)	H19A—C19—H19B	109.5
O21^i^—Yb2—O13	91.86 (11)	C18—C19—H19C	109.5
O17—Yb2—O13	86.55 (11)	H19A—C19—H19C	109.5
O21^i^—Yb2—O20	107.46 (11)	H19B—C19—H19C	109.5
O17—Yb2—O20	85.43 (11)	C18—C20—H20A	120.0
O13—Yb2—O20	147.43 (11)	C18—C20—H20B	120.0
O21^i^—Yb2—O15	78.21 (12)	H20A—C20—H20B	120.0
O17—Yb2—O15	78.83 (12)	N1—C21—C22	123.8 (4)
O13—Yb2—O15	74.24 (11)	N1—C21—H21	118.1
O20—Yb2—O15	134.39 (13)	C22—C21—H21	118.1
O21^i^—Yb2—O19	72.77 (11)	C21—C22—C23	118.8 (4)
O17—Yb2—O19	130.18 (11)	C21—C22—H22	120.6
O13—Yb2—O19	84.25 (11)	C23—C22—H22	120.6
O20—Yb2—O19	77.13 (12)	C22—C23—C24	117.3 (4)
O15—Yb2—O19	143.07 (12)	C22—C23—C26	121.7 (4)
O21^i^—Yb2—O22	79.59 (11)	C24—C23—C26	121.0 (4)
O17—Yb2—O22	86.73 (12)	C25—C24—C23	119.9 (4)
O13—Yb2—O22	140.30 (11)	C25—C24—H24	120.0
O20—Yb2—O22	70.56 (12)	C23—C24—H24	120.0
O15—Yb2—O22	66.07 (11)	N1—C25—C24	122.8 (4)
O19—Yb2—O22	128.18 (10)	N1—C25—H25	118.6
O21^i^—Yb2—O18	125.10 (11)	C24—C25—H25	118.6
O17—Yb2—O18	76.73 (12)	C30—C26—C27	116.8 (4)
O13—Yb2—O18	73.01 (11)	C30—C26—C23	119.8 (4)
O20—Yb2—O18	74.43 (11)	C27—C26—C23	123.5 (4)
O15—Yb2—O18	139.90 (10)	C28—C27—C26	119.7 (4)
O19—Yb2—O18	53.76 (10)	C28—C27—H27	120.2
O22—Yb2—O18	142.24 (11)	C26—C27—H27	120.2
O21^i^—Yb2—H222	78.36 (9)	N2—C28—C27	123.1 (4)
O17—Yb2—H222	82.96 (9)	N2—C28—H28	118.4
O13—Yb2—H222	123.21 (8)	C27—C28—H28	118.4
O20—Yb2—H222	86.98 (9)	N2—C29—C30	123.8 (4)
O15—Yb2—H222	48.96 (8)	N2—C29—H29	118.1
O19—Yb2—H222	140.72 (7)	C30—C29—H29	118.1
O22—Yb2—H222	17.13 (7)	C29—C30—C26	119.6 (4)
O18—Yb2—H222	153.31 (7)	C29—C30—H30	120.2
O7—Zn1—O9	125.99 (15)	C26—C30—H30	120.2
O7—Zn1—O11	112.47 (14)	O13—C31—O12	123.3 (4)
O9—Zn1—O11	112.01 (15)	O13—C31—C32	120.2 (4)
O7—Zn1—N1	99.31 (14)	O12—C31—C32	116.5 (4)
O9—Zn1—N1	99.50 (14)	C34—C32—C33	123.7 (5)
O11—Zn1—N1	102.42 (14)	C34—C32—C31	119.2 (5)
O14—Zn2—O16	120.46 (15)	C33—C32—C31	117.1 (4)
O14—Zn2—O12	117.56 (14)	C32—C33—H33A	109.5
O16—Zn2—O12	111.17 (14)	C32—C33—H33B	109.5
O14—Zn2—N2	95.41 (13)	H33A—C33—H33B	109.5
O16—Zn2—N2	105.31 (13)	C32—C33—H33C	109.5
O12—Zn2—N2	102.77 (14)	H33A—C33—H33C	109.5
Yb1—O1—H11	114.0 (2)	H33B—C33—H33C	109.5
Yb1—O1—H12	116.1 (3)	C32—C34—H34A	120.0
H11—O1—H12	120.6 (3)	C32—C34—H34B	120.0
C1—O2—Yb1	92.0 (3)	H34A—C34—H34B	120.0
C1—O3—Yb1	94.9 (3)	O15—C35—O14	125.5 (4)
C5—O4—Yb1	93.2 (3)	O15—C35—C36	118.9 (4)
C5—O5—Yb1	92.4 (3)	O14—C35—C36	115.6 (4)
C9—O6—Yb1	145.4 (3)	C38—C36—C37	124.0 (5)
C9—O7—Zn1	129.3 (3)	C38—C36—C35	118.9 (5)
C13—O8—Yb1	139.5 (3)	C37—C36—C35	117.2 (5)
C13—O9—Zn1	117.3 (3)	C36—C37—H37A	109.5
C17—O10—Yb1	163.9 (3)	C36—C37—H37B	109.5
C17—O11—Zn1	113.7 (3)	H37A—C37—H37B	109.5
C31—O12—Zn2	119.1 (3)	C36—C37—H37C	109.5
C31—O13—Yb2	141.7 (3)	H37A—C37—H37C	109.5
C35—O14—Zn2	129.3 (3)	H37B—C37—H37C	109.5
C35—O15—Yb2	147.5 (3)	C36—C38—H38A	120.0
C39—O16—Zn2	115.9 (3)	C36—C38—H38B	120.0
C39—O17—Yb2	171.8 (3)	H38A—C38—H38B	120.0
C43—O18—Yb2	91.0 (3)	O17—C39—O16	123.7 (4)
C43—O19—Yb2	94.7 (3)	O17—C39—C40	119.6 (4)
C47—O20—Yb2	127.2 (3)	O16—C39—C40	116.8 (4)
C47—O21—Yb2^i^	176.6 (3)	C42—C40—C41	124.8 (5)
Yb2—O22—H221	120.8 (3)	C42—C40—C39	118.1 (5)
Yb2—O22—H222	103.1 (2)	C41—C40—C39	117.1 (4)
H221—O22—H222	107.8 (4)	C40—C41—H41A	109.5
C21—N1—C25	117.4 (3)	C40—C41—H41B	109.5
C21—N1—Zn1	120.8 (3)	H41A—C41—H41B	109.5
C25—N1—Zn1	121.4 (3)	C40—C41—H41C	109.5
C28—N2—C29	117.0 (3)	H41A—C41—H41C	109.5
C28—N2—Zn2	126.5 (3)	H41B—C41—H41C	109.5
C29—N2—Zn2	116.6 (3)	C40—C42—H42A	120.0
O3—C1—O2	118.7 (4)	C40—C42—H42B	120.0
O3—C1—C2	120.3 (4)	H42A—C42—H42B	120.0
O2—C1—C2	121.0 (4)	O18—C43—O19	120.5 (4)
C4—C2—C3	123.7 (6)	O18—C43—C44	119.9 (4)
C4—C2—C1	117.3 (6)	O19—C43—C44	119.6 (4)
C3—C2—C1	119.0 (6)	C46—C44—C45	123.3 (5)
C2—C3—H3A	120.0	C46—C44—C43	118.2 (5)
C2—C3—H3B	120.0	C45—C44—C43	118.5 (5)
H3A—C3—H3B	120.0	C44—C45—H45A	109.5
C2—C4—H4A	109.5	C44—C45—H45B	109.5
C2—C4—H4B	109.5	H45A—C45—H45B	109.5
H4A—C4—H4B	109.5	C44—C45—H45C	109.5
C2—C4—H4C	109.5	H45A—C45—H45C	109.5
H4A—C4—H4C	109.5	H45B—C45—H45C	109.5
H4B—C4—H4C	109.5	C44—C46—H46A	120.0
O5—C5—O4	119.6 (4)	C44—C46—H46B	120.0
O5—C5—C6	120.1 (4)	H46A—C46—H46B	120.0
O4—C5—C6	120.3 (4)	O21—C47—O20	123.7 (4)
C7—C6—C8	124.0 (6)	O21—C47—C48	118.0 (4)
C7—C6—C5	118.9 (5)	O20—C47—C48	118.3 (4)
C8—C6—C5	117.1 (5)	C50—C48—C49	122.2 (5)
C6—C7—H7A	120.0	C50—C48—C47	119.4 (5)
C6—C7—H7B	120.0	C49—C48—C47	118.3 (5)
H7A—C7—H7B	120.0	C48—C49—H49A	109.5
C6—C8—H8A	109.5	C48—C49—H49B	109.5
C6—C8—H8B	109.5	H49A—C49—H49B	109.5
H8A—C8—H8B	109.5	C48—C49—H49C	109.5
C6—C8—H8C	109.5	H49A—C49—H49C	109.5
H8A—C8—H8C	109.5	H49B—C49—H49C	109.5
H8B—C8—H8C	109.5	C48—C50—H50A	120.0
O6—C9—O7	124.3 (4)	C48—C50—H50B	120.0
O6—C9—C10	118.8 (4)	H50A—C50—H50B	120.0

Symmetry codes: (i) −*x*, −*y*+1, −*z*.

### Hydrogen-bond geometry (Å, °)

**Table:**

*D*—H···*A*	*D*—H	H···*A*	*D*···*A*	*D*—H···*A*
O1—H11···O4^ii^	0.93	2.01	2.795 (4)	142
O1—H12···O2^ii^	0.72	2.30	2.903 (5)	142
O22—H221···O19^i^	0.71	2.06	2.758 (4)	169
C11—H11B···O6	0.93	2.44	2.758 (7)	100
C16—H16B···O9	0.96	2.39	2.776 (10)	104
C19—H19A···O3	0.96	2.54	3.339 (8)	141
C22—H22···O18^iii^	0.93	2.45	3.297 (5)	152
C24—H24···O5^iv^	0.93	2.52	3.435 (6)	169
C27—H27···O18^iii^	0.93	2.40	3.330 (5)	175
C29—H29···O14	0.93	2.47	3.020 (5)	118
C30—H30···O5^iv^	0.93	2.42	3.256 (6)	149
C34—H34B···O13	0.93	2.46	2.772 (7)	100
C42—H42B···O17	0.93	2.40	2.726 (7)	100
C49—H49B···O20	0.96	2.40	2.772 (7)	103
C50—H50B···O13^i^	0.93	2.59	3.521 (8)	177

Symmetry codes: (ii) −*x*+1, −*y*−1, −*z*+3; (i) −*x*, −*y*+1, −*z*; (iii) −*x*, −*y*, −*z*+1; (iv) −*x*+1, −*y*, −*z*+2.
